# HIV associated Lymphocytic Interstitial Pneumonia: a clinical, histological and radiographic study from an HIV endemic resource-poor setting

**DOI:** 10.1186/s12890-015-0030-2

**Published:** 2015-04-22

**Authors:** Richard N van Zyl-Smit, Jashira Naidoo, Helen Wainwright, Quanita Said-Hartley, Malika Davids, Hillel Goodman, Sean Rogers, Keertan Dheda

**Affiliations:** Division of Pulmonology & UCT Lung Institute, Department of Medicine, Lung Infection and Immunity Unit, University of Cape Town, Cape Town, South Africa; Department of Anatomical Pathology, UCT Faculty of Health Sciences & NHLS Laboratories, Groote Schuur Hospital, Cape Town, South Africa; Department of Radiology, Groote Schuur Hospital, Cape Town, South Africa; Constantiaberg Hospital, Cape Town, South Africa; Institute of Infectious Diseases and Molecular Medicine, University of Cape Town, Cape Town, South Africa

**Keywords:** Lymphocytic interstitial pneumonia, HIV, Tuberculosis, Histology

## Abstract

**Background:**

There is a paucity of clinical and histopathological data about HIV-associated lymphocytic interstitial pneumonitis (LIP) in adults from HIV endemic settings. The role of Ebstein-Barr virus (EBV) in the pathogenesis remains unclear.

**Methods:**

We reviewed the clinical, radiographic and histopathological features of suspected adult LIP cases at the Groote Schuur Hospital, Cape Town South Africa, over a 6 year period. Archived tissue sections were stained for CD3, CD4, CD8, CD20 and LMP-1 antigen (an EBV marker).

**Results:**

42 cases of suspected LIP(100% HIV-infected) were identified. 75% of patients were empirically treated for TB prior to being referred to the chest service for further investigation. Tissue samples were obtained using trans-bronchial biopsy. 13/42 were classified as definite LIP (lymphocytic infiltrate with no alternative diagnosis), 19/42 probable LIP (lymphocytic infiltrate but evidence of anthracosis or fibrosis) and 10 as non-LIP (alternative histological diagnosis). Those with definite LIP were predominantly young females (85%) with a median CD4 count of 194 (IQR 119–359). Clinical or radiological features had poor predictive value for LIP. Histologically, the lymphocytic infiltrate comprised mainly B cells and CD8 T cells. The frequency of positive EBV LMP-1 antigen staining was similar in definite and non- LIP patients [(2/13 (15%) vs. 3/10 (30%); p = 0.52].

**Conclusions:**

In a HIV endemic setting adult HIV-associated LIP occurs predominantly in young women. The diagnosis can often be made on transbronchial biopsy and is characterized by a predominant CD8 T cell infiltrate. No association with EBV antigen was found.

## Background

Lymphocytic interstitial pneumonia (LIP) is an uncommon histopathologic entity characterized by infiltration of the interstitium and alveolar spaces of the lung by lymphocytes, plasma cells, and other lymphoreticular elements [1]. It was initially described in 1966, prior to the HIV era, by Carrington and Liebow [2] and remains a rare form of interstitial lung disease in HIV uninfected adults [3]. With the global spread of HIV, LIP has become a more common particularly in HIV endemic countries. However, there are hardly any data about the clinical and histological characteristics of HIV-associated LIP in adults [4,5], and particularly those from HIV endemic countries.

Although the aetiology of LIP remains unclear an association with several autoimmune disorders including Sjogrens syndrome, systemic lupus erythematous (SLE), rheumatoid arthritis and pernicious anaemia is well recognized [3]. In HIV-associated LIP a possible etiological factor includes HIV-induced proliferation of bronchus-associated lymphoid tissue BALT [6,7]. An alternative hypothesis implicates EBV infection as an agent important in the pathogenesis of LIP in HIV uninfected persons [8]. However, this hypothesis has not been investigated in the context of HIV-associated LIP.

The clinical (non-productive cough, progressive dyspnoea, and crackles) [1,5,9,10] and radiological features (ground-glass appearance, centrilobular nodules, and interstitial thickening) of LIP, using both chest x-ray and CT, are non-specific [11-14]. Thus, open lung biopsy is often required to clarify the diagnosis and to exclude other possibilities including TB, which may often present with similar features. However, open lung biopsy is often unavailable or inaccessible in resource poor settings. The role and utility of bronchoscopy and transbronchial biopsy as an alternative diagnostic approach remains unclear.

Thus, there remains a paucity of data on HIV-associated LIP from high burden settings to guide clinical practice, and very little is known about the optimal diagnostic approach, clinical outcomes, and histological appearance of HIV-associated LIP. We therefore retrospectively reviewed the clinical, radiological, and histopathological features of all cases of suspected LIP seen at our institution, a tertiary referral centre, in Cape Town, South Africa, over a 6-year period. We further interrogated tissue sections to determine whether EBV co-infection may have a role in the pathogenesis of HIV-associated LIP.

## Methods

The study was conducted in adults only, at the E16 Respiratory Clinic at Groote Schuur Hospital in Cape Town, South Africa, which maintains a comprehensive database of all bronchoscopic procedures and patient diagnoses. Study approval was obtained from the University of Cape Town Health Sciences Faculty Research Ethics Committee. Written informed consent was not obtained as the study was a retrospective chart and case review only.

### Case identification

**Figure 1:**
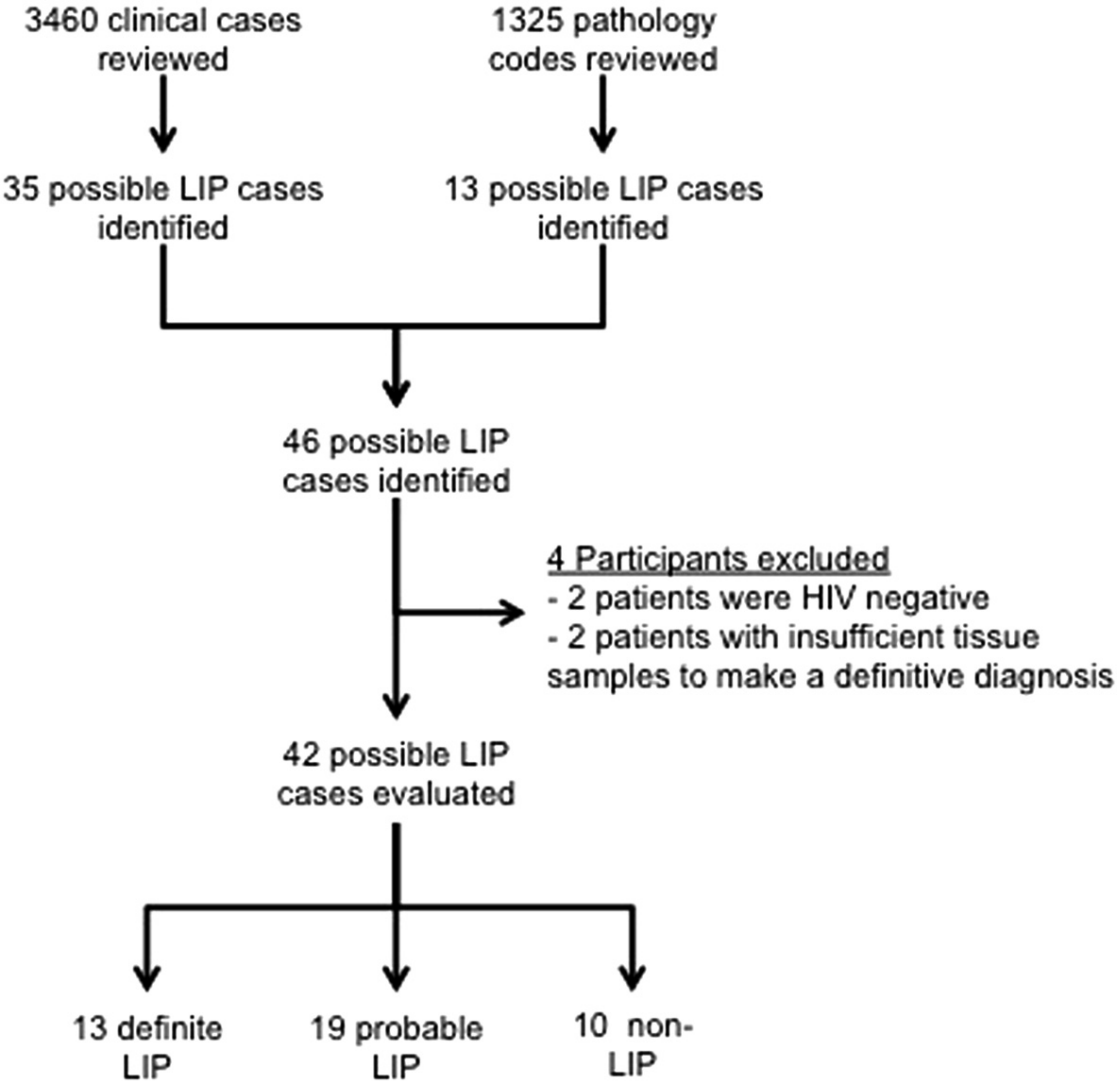
Study overview.

All clinical bronchoscopy case records were reviewed over a 6-year period from 2003 to 2008. In parallel, and to minimize ascertainment bias, all cases of LIP reported by the Department of Pathology from the Groote Schuur Hospital were identified. Thus, a total of 1325 pathology codes covering all lung pathological diagnoses were reviewed for the same time period to identify additional cases not identified from the respiratory service records. All case records where LIP was considered in the differential diagnosis were extracted for review (see Figure 1 for a study overview). Only HIV-infected patients and cases where a tissue biopsy was available to ascertain the cause of the underlying lung disease were finally included in the analysis.

### Data collection and review

Patient demographics, clinical history, presenting signs and symptoms and lung function testing data (basic spirometry where available) were extracted from the clinical notes. Two senior thoracic radiologists independently reviewed radiological studies including chest X-ray and CT scans. (HG, QS) Radiological features were classified into several pre-specified categories: reticular/nodular pattern, presence of ground glass or alveolar infiltrate, pleural effusions and lymphadenopathy. Discordance was resolved by a third reader (senior pulmonologist).

The classification of LIP disease was made on histology by an independent senior pulmonary pathologist (HW) who was blinded to all clinical information. The histological definition of LIP used for disease classification was the presence of: expanded/thickened alveolar walls by large number of small lymphocytes & plasma cells (not in alveolar spaces) most marked around broncho-vascular spaces and around vessels/lymphatics, and frequent lymphoid follicles in the absence of an alternative disease process associated with a lymphocytic infiltrate. A diagnosis of probable LIP was made where the lymphoid infiltrate was accompanied by second process such as anthracosis or fibrosis. Thus, the diagnosis of LIP was likely but the presence of a confounding disease process could not be excluded with certainty. Additionally, the specimen was considered adequate and sufficient tissue present if it contained a bronchiole (to evaluate follicular hyperplasia) and adequate surrounding lung parenchyma. A diagnosis of non-LIP was made where clear cut features of alternative disease process was present. Additional immune-histochemical staining was performed using antibodies for CD4 (Clone 4B12 Leika), CD8 (Clone NCL-CD8-4B11 Leika), CD20 (Clone L26 Dako) and EBV – LMP-1 Antigen (Clone CS.1-4 Dako).

### Statistical analysis

Categorical variables were compared using the χ^2^ test and continuous variables were compared using Student’s t-test where appropriate. The Mann–Whitney test was used for comparison of non-parametrically distributed continuous variables. (Graphpad Prism, Version 5.03 and Open Epi, Version 2.3.1 were used).

## Results

### Case identification

A total of 3460 clinical bronchoscopy case records were reviewed and 33 possible LIP cases were identified (see Figure 1 for study overview). A further 13 potential cases were identified from the pathological biopsy records. Of the 46 potential cases identified, 4 were excluded (Figure 1).

### Patient classification and demographic features

Of the 42 patients available for evaluation, 13 were classified as definite LIP (lymphocytic infiltrate with no alternative diagnosis), 19 probable LIP (lymphocytic infiltrate but evidence of anthracosis or fibrosis) and 10 as non-LIP (alternative histological diagnosis). All participants were HIV-infected, and only one patient with LIP was on anti retroviral therapy at time of diagnosis. The LIP patients were predominantly female 85%(11/13), young (median age of 34 years) with severe immunosuppression (median CD4 count 194 cells/ml); see Table 1 for details and p values.

**Table 1:** **Clinical characteristics of patients with suspected LIP**

	**Definite LIP**	**Non-LIP**	**Probable LIP**	**Definite & probable LIP**	**P value**
	**n = 13**	**n = 10**	**n = 19**	**n = 32**	
**Demographic characteristics**					
Age median (range)	34 (21–57)*	42 (26–66)*	39(20–52)	35 (20–57)	*p = 0.11
Gender: Female n (%)	11(85%)*	3 (30%)*^#^	15 (79%)	20 (77%)^#^	*p = 0.006
					^#^p = 0.008
CD4 count median (range)	194 (104–464)*	157(51–323)*	216 (30–444)	216 (30–464)	p = 0.18
Number of participants on ARV at presentation	1/11 (9%)	5/8 (62.5%)*	8/13 (61.5%)	9/24(37.5%)*	*p = 0.25
**Symptoms**					
Duration in months Median (range)	5 (1–84)	4 (.75-12)	6 (0.5-24)	6 (0.5-84)	p = 0.45
Presence of cough	10/11 (90%)	10/10 (100%)	16/18 (88%)	26/29(89.7%)	
Productive cough	7/11 (64%)*	6/10(60%)*	12/16 (75%)	19/27 (71%)	*p = 0.43
Dyspnea (NHYA)					
Class 1	3 (25%)	2 (20%)	6 (33%)	9 (30%)	
Class 2	8 (67%)	4 (40%)	9 (50%)	17 (57%)	
Class 3	1(3%)	3 (30%)	3 (17%)	4 (13%)	
Class 4	0	1 (10%)	0(0%)	0	
Referral diagnosis of non-resolving TB	77%	70%	86%		
**Examination findings**					
BMI kg/cm^2^ mean (SD)	25.3(5.3)	23.8(6.2)	23.9(5.4)	24.6(5.2)	p = 0.94
Presence of clubbing	0/ 11 (0%)	3/10 (30%)	6/18(33%)	6/29 (20%)	
**Auscultation**					
Normal	4 (33%)	2 (20%)	7 (39%)	11 (38%)	
Bibasilar crackles	7 (58%)	6 (60%)	9 (50%)	16 (55%)	
Diffuse crackles	0	1(10%)	2 (11%)	2 (7%)	
Wheeze	0	0	0	0	
Bronchial breathing	1 (8%)	1(10%)	0	0	

*,^#^p value for comparison between groups indicated.

### Clinical features

The predominant symptom was cough 90% (10/11) which was productive in 64% (7/11) of participants. The median duration of symptoms was 5 months (range 1–84) with class II or III dyspnea occurring in 70%(9/13) of patients. Notably, the most common referring diagnosis in those confirmed to have LIP (77%) was non-resolving tuberculosis. Clinical features were varied with a normal chest examination in a third of LIP patients. No participants with LIP had digital clubbing. There were no clinical features that distinguished LIP from the non-LIP group (Table 1).

### Lung function

**Figure 2:**
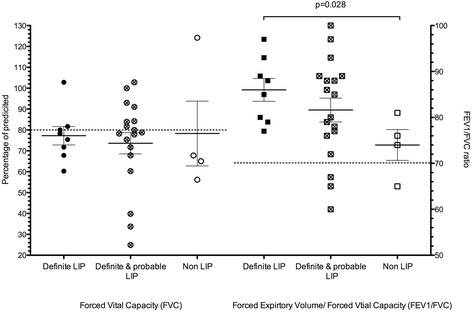
Lung function abnormalities in patients with LIP. Individual patient values are depicted with Mean and SEM. Significant comparisons are as indicated with p values. FVC percentage predicted is depicted on left axis. FEV1/FVC ratio is depicted on the right axis. LIP(Lymphocytic interstitial pneumonia). Horizontal dotted lines indicated normal cut off values.

Lung function tests were only available for 8/13 definite LIP patients. Reduced volumes (restrictive lung function) were evident for most participants with LIP (6/8); median percentage predicted FVC was 77% (range 60–103), FEV1 64% (52–100), and FEV1/FVC ratio 87% (range 77–97). None of the 8 definite LIP patients had obstructive lung disease. The lung function abnormalities in the probable LIP group were similar to the LIP group (Figure 2).

**Figure 3:**
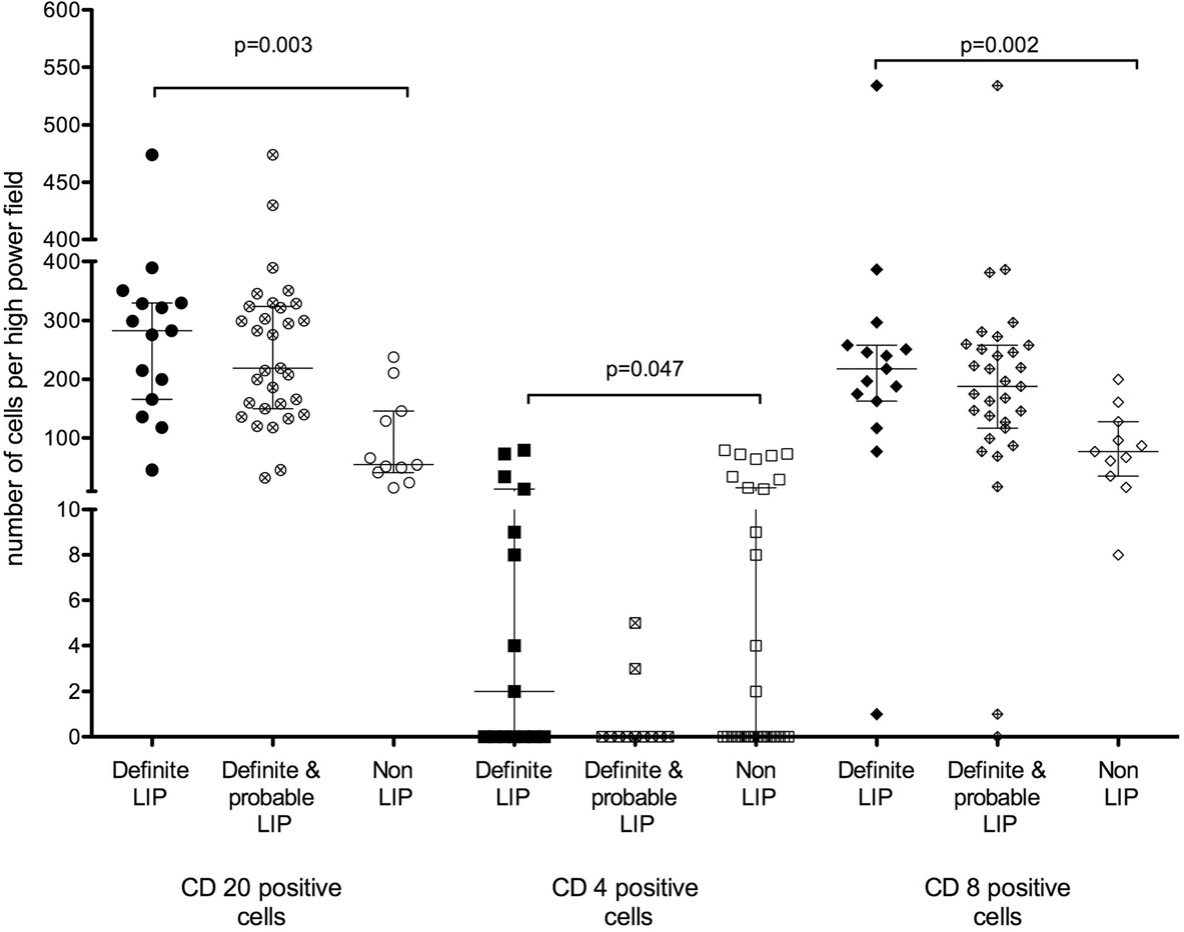
Immunomarkers for B and T cells in patients with confirmed LIP compared to non- LIP patients. Individual patient data is depicted with Median and Interquartile range. Comparisons between LIP and Non LIP are depicted with p values.

### Radiology

38 participants had chest x-rays and 14 had CT scans available for evaluation (see Table 2). No chest x-rays were considered normal in the LIP group. Almost half (42%) of the participants had a reticular or reticular nodular infiltrate. Hilar lymphadenopathy was present in 50% (6/12). Pleural effusions were noted in 2 participants. There were no distinguishing chest x-ray features between the diagnostic groups. Six CT scans were available for analysis in the definite LIP group. Two demonstrated a ground glass pattern and 3 had nodular infiltrates. No interlobular septal thickening, bronchiectasis, thickened bronchovascular bundles, lymph nodes or pleural effusions were seen in the definite LIP group and only 1 patient had lung cysts.

**Table 2:** **Radiological findings based on chest x-rays available from 38 patients**

	**Definite LIP**	**Non-LIP**	**Probable LIP**	**Definite & probable LIP**	**P value**
	**N = 12**	**N = 8**	**18**	**30**	
**Dominant CXR pattern**					
Normal	0	0	1 (6%)	1 (3%)	
Reticular/reticulo-nodular	5 (42%)	4 (50%)	9 (50%)	14 (47%)	
Alveolar	4 (33%)	1 (13%)	1 (11%)	5 (17%)	
Ground Glass	3 (25%)	3 (38%)	7 (44%)	10 (33%)	
Pleural Effusion	2 (17%)	0	2 (11%)	4 (13%)	
Lymphadenopathy	6 (50%)*	2 (25%)*	5 (28%)	11 (37%)	*p = 0.15

*p value for comparison between groups indicated.

### Histological diagnosis

Of the 46 cases reviewed 4 were excluded as two were found to be from HIV negative participants and a further 2 had insufficient tissue to perform the additional stains. 13 cases met the case definition of LIP with 19 having additional features suggesting dual or alternative pathology.

Using additional staining for B cell and T cell markers, the cellular predominance was shown to be of CD8 cell origin. There were also high numbers of CD20 positive B cells and high CD8 to CD4 ratio (Figure 3). Only 2 patients with definite LIP had EBV positive biopsies. There was no difference in the frequency of EBV positive staining between the LIP (15%) and the non-LIP (30%) group (p = 0.52), data not shown.

## Discussion

There are virtually no data about HIV-associated LIP from HIV endemic settings. Our data suggest that HIV-associated LIP although still an uncommon diagnosis in high HIV burden settings, is frequently misdiagnosed as TB. Clinical and radiological presentation is non-specific. The diagnosis, however, can be made with adequate transbronchial biopsies rather than resorting to open lung biopsy and the cellular phenotype is that of CD8 and CD20 predominance. In this preliminary study the LMP-1 EBV antigen does not appear to be important in the pathogenesis of HIV-associated LIP.

It is striking that 75% of patients were initially treated for TB, which reflects the high burden of TB in South Africa [15]. Furthermore the lack of access to diagnostic tools to exclude alternative diagnoses makes empiric therapy a common practice [16]. Thus, in a high TB/HIV burden settings, the symptoms and CXR features of LIP are frequently attributed to TB and the diagnosis is generally missed unless a high index of suspicion and adequate follow up of patients is maintained. Even with the advent of Gene Xpert MTB/RIF testing now being available in several TB endemic countries, symptomatic patients with advanced HIV will most likely be treated for TB prior to referral to a tertiary level service for a bronchoscopy [15,17].

The demographic characteristics and clinical features of this HIV-associated LIP cohort of patients is similar to that described in the literature: the preponderance of young females has been described previously [1,3,13] although it has been also been described in white men [18]. The prevalence of HIV in South Africa is highest amongst black African women and thus may be reflected in the demographics of our population sample. HIV-associated LIP is generally not associated with advanced HIV in most published studies and case reports where CD4 counts are generally reported to be in the normal range [18-20]. In our cohort, although the median CD4 count was 194, several participants had CD4 counts over 300. In this cohort, and not dissimilar to other reports [9], a wide duration of symptoms prior to diagnosis was recorded; the median duration of symptoms of 5 months suggests that an acute presentation is not common. Symptoms and examination findings are also generally non-specific, with cough and dyspnea being the hallmark, as previously reported in the HIV uninfected persons with LIP [1,3].

In keeping with other literature, lung function tests showed a restrictive pattern [13]. Unfortunately as these participants were being investigated for infectious lung diseases, full lung volumes and diffusing capacity was not measured. The radiographic features were predominantly of an interstitial pattern with associated lymphadenopathy. In contrast to the published literature, pleural effusions occurred in roughly 15% of participants. CT scans did not show any predominant pattern that could potentially assist with the diagnosis.

There were several insights from the histopathological analysis. Although CD8 preponderance has been well described in LIP the type of CD4 versus CD8 infiltration in the context of HIV-associated LIP has not been well studied nor described. In addition we evaluated the possible role of EBV in the pathogenesis of HIV-associated LIP. Several pathogenic mechanisms have been described including autoimmunity, HIV-associated lymphocytosis, dysfunctional regression of an immunological response to antigen in the lung, and viral infections including EBV [3,4]. Data on EBV have been discordant [21-23]. EBV latent membrane protein 1 is an attractive hypothesis as it up-regulates bcl-2, which has been shown to confer a survival advantage for lymphocytes in other lymphoproliferative disorders, and thus could enhance tissue lymphocyte accumulation and survival in LIP. However, in contradistinction to Barbera *et al.* who detected EBV in the open-lung biopsy specimens of 9 of 14 patients with LIP [24] and another study that detected EBV DNA in 80% of lung specimens from HIV-positive children with LIP [21], we failed to detect any difference in EBV frequency using an LMP-1 immunoassay.

This study has several limitations. The small number of subjects and retrospective design lends itself to ascertainment bias; however, we did compare LIP to participants with non-LIP. There were also missing data. However, we do show that it is possible to make the diagnosis using transbronchial biopsy and have confirmed the CD8 predominant nature of infiltrates in HIV-infected persons. We did not evaluate other EBV-associated antigens or use PCR however the lack of detectable antigen in most biopsies makes EBV an unlikely culprit driving pathogenesis in the African setting, and presence of DNA may often constitute an epiphenomenon rather than causality.

## Conclusion

In conclusion, although HIV-associated LIP remains an uncommon condition, even in HIV-endemic settings, the diagnosis can often be made via bronchoscopy and transbronchial biopsy. The disease occurs in Africa predominantly in young women with moderate to severe immunosuppression. The histological features are typically of a CD8 and CD20 infiltrate and EBV does not appear to be a major pathogenic feature in this adult population.

## Abbreviations

HIV: Human immunodeficiency virus
LIP: Lymphocytic interstitial pneumonitis
EBV: Ebstein-Barr virus
